# POCUS-guided suprapubic bladder aspiration in children: a systematic review and meta-analysis

**DOI:** 10.1590/0100-3984.2025.0100

**Published:** 2026-09-08

**Authors:** Bianca S. Romeo, Samuel Menezes, Eduardo F. Muricy, Rafael G. S. Barreto, Lucas B. S. Carvalho, Gabriela M. M. D. Melo, Maria F. F. Viana, Jennifer R. Marin, Rodrigo C. V. Duarte

**Affiliations:** 1 Department of Medicine, Bahiana School of Medicine and Public Health, Salvador, BA, Brazil; 2 Department of Medical Sciences, Federal University of Bahia, Salvador, BA, Brazil; 3 Departments of Pediatrics, Emergency Medicine, and Radiology, University of Pittsburgh School of Medicine, Pittsburgh, PA, USA; 4 Zarns School of Medicine, Salvador, BA, Brazil

**Keywords:** Point-of-care systems, Ultrasonography, Urine specimen collection, Infant, Infant newborn., Sistemasautomatizadosde assistência junto ao leito, Ultrassonografia, Coleta de urina, Lactente, Recém-nascido.

## Abstract

**Objective:**

Point-of-care ultrasound (POCUS) has been employed to assist with invasive techniques, such as suprapu-bic aspiration (SPA). We aimed to conduct a meta-analysis to compare the effectiveness of suprapubic bladder aspira-tion in children, with and without the use of POCUS.

**Methods:**

We conducted a systematic search across the MEDLINE, EMBASE and Cochrane Central Register of Con-trolled Trials databases for articles published through February 2025. Eligible studies included randomized controlled trials (RCT) comparing SPA with and without POCUS in pediatric populations. Data extraction focused on overall pro-cedural success rates, as well as rates of success at first and second attempts.

**Results:**

We analyzed seven trials collectively evaluating 468 suprapubic bladder aspirations. POCUS-guided SPA showed higher overall success rates (OR = 4.88; 95% CI: 2.36-10.09; *p* < 0.0001; I^^2^^ = 10%). Although no significant difference was found in first-attempt success, POCUS improved success within two attempts (OR = 7.75; 95% CI: 1.54-39.09; *p* = 0.01; I^^2^^ = 61%), although without significantly reducing the mean number of attempts. However, sensi-tivity analysis showed a benefit after the exclusion of one outlier study.

**Conclusions:**

POCUS improves SPA success in children and may enhance sterile urine collection, although training and equipment access remain challenges.

## Abstract (pt)

**Objetivo:**

A ultrassonografia à beira-leito (POCUS) é uma ferramenta utilizada para auxiliar em técnicas invasivas, como a aspiração suprapúbica (ASP). O objetivo deste estudo foi realizar uma meta-análise para comparar a efetivi-dade da aspiração vesical suprapúbica em crianças com e sem o uso de POCUS.

**Métodos:**

Foi realizada uma busca sistemática nas bases de dados MEDLINE, EMBASE e Cochrane (CENTRAL) até fevereiro de 2025. Foram incluídos ensaios clínicos randomizados que compararam a ASP com e sem o uso de POCUS em populações pediátricas. A ext-ração de dados concentrou-se nas taxas de sucesso do procedimento em geral e nas primeiras e segundas tentativas.

**Resultados:**

Sete estudos (n = 468) foram incluídos na análise. A ASP guiada por POCUS apresentou maiores taxas de sucesso geral (OR = 4,88; IC 95%: 2,36-10,09; *p* < 0,0001; I^^2^^ = 10%). Não houve diferença significativa no sucesso à primeira tentativa, porém o sucesso em até duas tentativas foi maior (OR = 7,75; IC 95%: 1,54-39,09; *p* = 0,01; I^^2^^ = 61%). O POCUS não reduziu significativamente o número médio de tentativas, embora a análise de sensibilidade tenha demonstrado benefício após a exclusão de um estudo discrepante.

**Conclusões:**

O uso do POCUS aumenta as taxas de sucesso da ASP em crianças e pode favorecer a obtenção de amostras urinárias estéreis, embora o treinamento e o acesso ao equipamento ainda representem desafios.

## INTRODUCTION

Urinary tract infections are one of the most common bacterial infections in children and the leading cause of bacterial infections in infants^**(^1^-^3^)**^. Ten percent of children experience at least one infection between 1 month and 6 years of age^**(^4^)**^. Although early diagnosis is important given the risk of long-term renal injury, obtaining a sterile urine specimen can be challenging, especially in children who are not toilet trained^**(^5^)**^. Urine collection, which can be performed by using noninvasive methods, such as a bag or clean-catch technique, or invasive methods, such as bladder catheterization or suprapubic aspiration (SPA), is essential for confirming the diagnosis^**(^6^)**^. However, each of those techniques has its limitations, particularly regarding contamination rates, which can lead to false positives^**(^7^)**^. Clinical guidelines generally recommend clean-catch sampling or SPA as preferred methods^**(^8^)**^, with SPA pre-senting the lowest contamination rates in urine sample collection, as well as exhibiting high sensitivity^**(^9^,^10^)**^.

Point-of-care ultrasound (POCUS) has become an important procedural tool in clinical practice, providing a noninvasive approach to assess bladder size and ana-tomical positioning prior to invasive techniques, such as SPA^**(^11^)**^. The use of POCUS facilitates visualization of the urinary bladder, aiding in localization and ensuring ad-equate urine sample collection^**(^12^)**^. The use of ultrasound can also increase urine collection by precisely locating a full bladder, providing real-time visualization during the procedure, which may reduce complications and improve the SPA success rate^**(^13^)**^. However, despite the increasing use of POCUS in multiple clinical contexts, there is a lack of consensus among studies evaluating the effectiveness of this tool for SPA, particularly for improving procedural success.

Although some single-center studies suggest that the use of POCUS improves SPA success, robust evi-dence of its superiority over the blind technique remains limited. Therefore, the aim of this systematic review and meta-analysis was to address this gap by comparing SPA performed with and without POCUS, in children. Specifically, we evaluated differences in success rates (overall and at first or second attempt), as well as the mean number of attempts required in order to obtain a successful sample, providing a critical analysis that may directly impact clinical practices for urine collection in children. By critically analyzing these outcomes, we hope to generate evidence to improve clinical practice for urine collection in pediatric patients.

## MATERIALS AND METHODS

This was a systematic review and meta-analysis, performed in accordance with the Cochrane Collabora-tion and the Preferred Reporting Items for Systematic Reviews and Meta-Analyses guidelines^**(^14^)**^. The proto-col for this review was registered in the International Prospective Register of Systematic Reviews (Identifier: CRD420250652095)

### Search strategy and inclusion criteria

A systematic digital search was conducted across the MEDLINE, EMBASE and Cochrane Central Register of Controlled Trials databases by two authors (S.M. and B.S.R.), working independently, up to February 2025. The search strategy included the terms “Ultrasonography”, “Ultrasound”, “Point of care ultrasonography”, “POCUS”, “Suprapubic aspiration”, “Bladder aspiration”, “Bladder puncture”, “Suprapubic puncture”, “Infant”, “Infants”, “Newborn”, “Neonate”, “Pediatrics”, “Pediatric”, “Child”, and “Children”. The complete search strategy can be found in Appendix 1. The same two researchers (B.S.R. and S.M.), working independently, screened titles and abstracts for eligibility, consulting a senior investigator in case of disagreements. Full texts, supplementary ma-terials, online appendices, and reference lists of eligible studies were reviewed to ensure compliance with the inclusion criteria and identify additional relevant stud-ies. Discrepancies were resolved through discussion with other reviewers until consensus was reached.

To be included, articles had to meet all of the follow-ing criteria: including data from prospective randomized controlled trials (RCTs); having enrolled pediatric pa-tients (< 24 months of age) who underwent SPA; having compared the SPA procedure with and without the use of POCUS; having been published as a full-text article. Observational studies that did not compare POCUS-guided SPA with traditional SPA were excluded.

### Data analysis and quality assessment

A comprehensive data extraction process was con-ducted by two investigators (E.F.M. and L.B.S.C.), work-ing independently, including population data such as the number of patients, mean age, gender distribution, mean weight, and indication for SPA. Specific information re-garding SPA was also collected, covering aspiration criteria when POCUS was used, materials for the procedure, the definition of a successful sample, and the ultrasound device employed. Each of the studies included defined “successful sample” differently, as detailed in Table 1. Definitions included aspiration of urine within up to three attempts, obtaining any amount of urine, requiring a minimum volume of 1 cc, or considering the bladder empty after aspiration. In three studies, the criteria for success were not explicitly stated. These variations were recorded during data extraction and were considered in the qualitative synthesis and sensitivity analyses. The main outcome collected was success rate, with success at first attempt, success up to second attempt and mean num-ber of attempts as secondary outcomes. The risk of bias was assessed by using the Cochrane risk of bias tool for randomized trials, designated the RoB 2 tool^(^14^)^. Potential bias was evaluated across five domains-randomization process, deviations from intended interventions, missing outcome data, measurement of the outcome, and selection of the reported result- each study thus being classified as having a high, low, or unclear risk of bias.

**Table 1:** Baseline characteristics of studies and patient populations.

Study	RCT Design	Country	Group							Ultrasound device used	Definition of successful sample collection	Reason for aspiration	Criteria for aspiration when POCUS was used	Materials used to perform aspiration
			POCUS		Control		All subjects							
			(n)	Age (months)	(n)	Age (months)	Age (months)	Male	Weight (kg)					
				Mean ± SD		Mean ± SD	Mean ± SD	(%)	Mean ± SD					
Choi et al.^(^18^)^	Single-center	South Korea	46	9.4 ± 0.7	32	9.7 ± 0.7	n/a	53.8	n/a	LOGIQ 400	Urine aspirated in up to 3 attempts	Suspected UTI	10 × 20 mm of urine in the bladder	23 G needle attached to a 10-cc syringe
Chu et al.**^(^21^)^**	Single-center	Hong Kong	30	5.2 ± 3.4	30	4.2 ± 3.1	4.7 ± n/a	45.0	n/a	Aloka 620	n/a	Fever and a urine sample testing positive for leukocyte esterase or nitrites, with or without leukocytes (>5 mm^3^) OR a positive bacterial culture in a previous bag urine sample	Bladder volume estimated to be greater than 3 mL	21 G needle
Gochman et _al._**(11)**	Single-center	USA	35	1.7 ± 2.8	31	2.3 ± 2.9	1.98 ± n/a	60.6	4.98 ± n/a	ScanMate II	Any amount of urine aspirated	Judgment of the examining physician	Anteroposterior and transverse maximum diameters ≥ 2 cm	22 G spinal needle attached to a 3-mL syringe
Kiernan et _al._**(17)**	Single-center	USA	28	n/a	25	n/a	n/a	n/a	n/a	Pie Medical Imaging	n/a	Determined by the clinical team	Bladder measured at least 1 × 1 cm (longitudinal and transverse views)	Sterile 23 G needle attached to a 3-mL syringe
Lee et al.**^(^19^)^**	Single-center	South Korea	32	n/a	28	n/a	5.28 ± n/a	70.0	7.23 ± n/a	Sonoace 8800	n/a	Suspected UTI	Urine present	21 G needle attached to a 10-mL syringe.
Mahdipour et al.**^(^20^)^**	Single-center	Iran	38	n/a	38	n/a	1.17 ± 0.47	59.6	4.19 ± 1.25	Ultrasonix	≥ 1 cc of urine aspirated	Suspected UTI	n/a	10-cc syringe with a 24 G needle
Munir et _al._**(12)**	Single-center	Australia	39	n/a	36	n/a	n/a	37.3	n/a	n/a	Bladder considered empty	Uncontaminated urine specimen required for microscopy and culture	10 ml of urine in the bladder	23 G needle attached to a 2- or 5-mL syringe

UTI, urinary tract infection.

### Statistical analysis

Odds ratios with 95% confidence intervals were com-bined using a random-effects model to evaluate treatment effects for categorical outcomes. Heterogeneity was as-sessed through the use of the Cochran Q test and calcu-lation of the I^^2^^ statistic, with significance defined as *p* < 0.10 and I^^2^^ > 25%, respectively. Statistical analyses were performed using the software Review Manager, version 5.4^**(^15^)**^ and R-Studio, version R-4.3.3^(^16^,^17^)^. A leave-one-out sensitivity analysis was conducted to determine the impact of individual studies on the overall meta-analysis results.

## RESULTS

### Study selection and baseline characteristics

**Figure 1:**
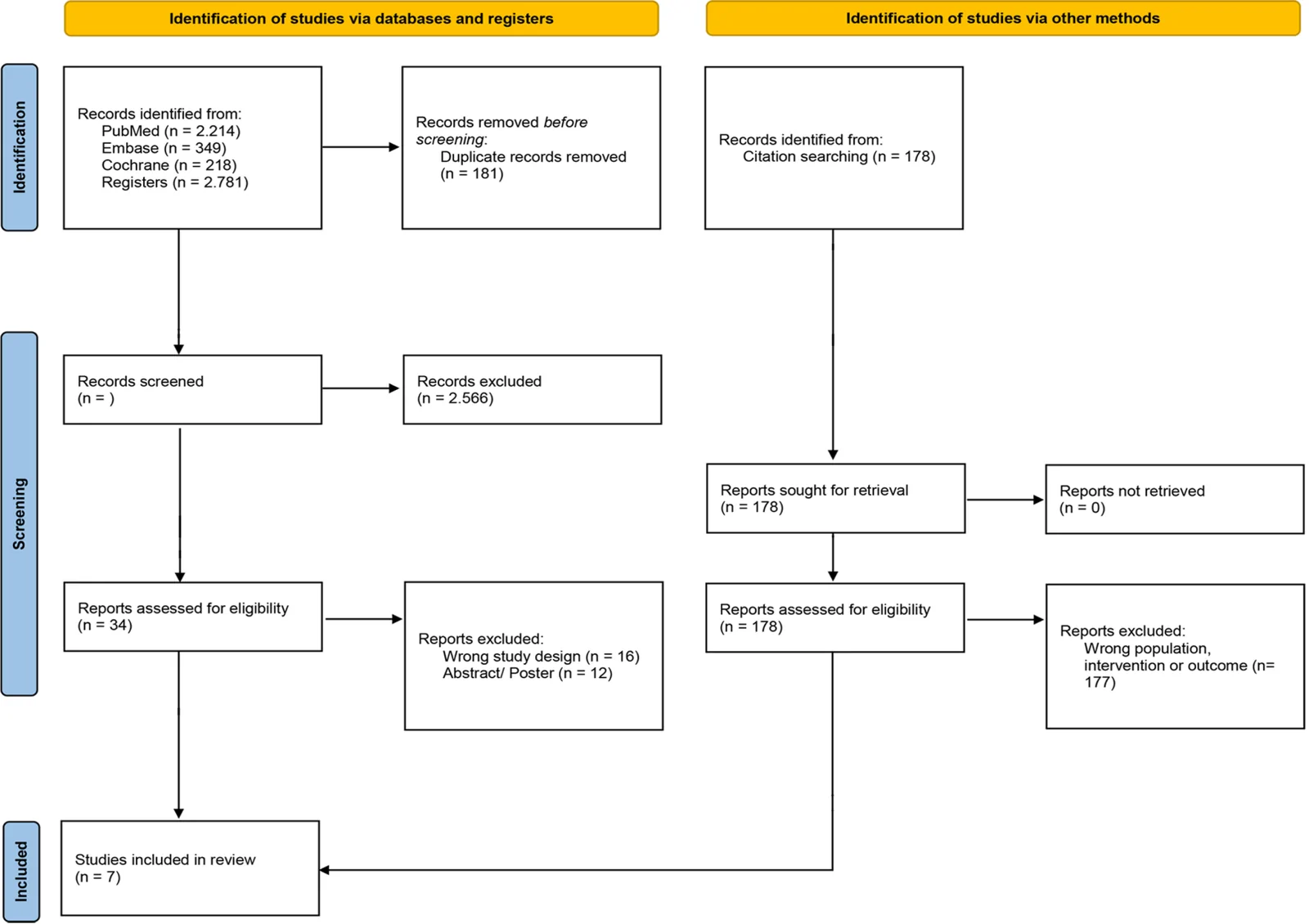
Flow chart of the article selection process, conducted in accordance with the Preferred Reporting Items for Systematic Reviews and Meta-Analyses recommendations.

The systematic search identified 2,781 studies; after duplicates had been removed, 2,600 remained. Through title and abstract screening, an additional 2,566 studies were removed. The remaining 34 studies underwent full-text evaluation, which resulted in the exclusion of 27 studies. Therefore, seven RCTs were included in the final analysis^**(^11^,^12^,^18^-^22^)**^. The screening and eligibility process is illustrated in Figure 1.

The studies included a collective total of 468 participants, with 248 allocated to the POCUS groups and 220 allocated to the control groups. The population consisted of neonates and infants, with mean ages ranging from 0.47 to 9.7 months and mean weights ranging from 1.25 to 7.23 kg. Among the seven studies included, age was reported in four and weight was reported in three.

### Success rates

**Figure 2:**
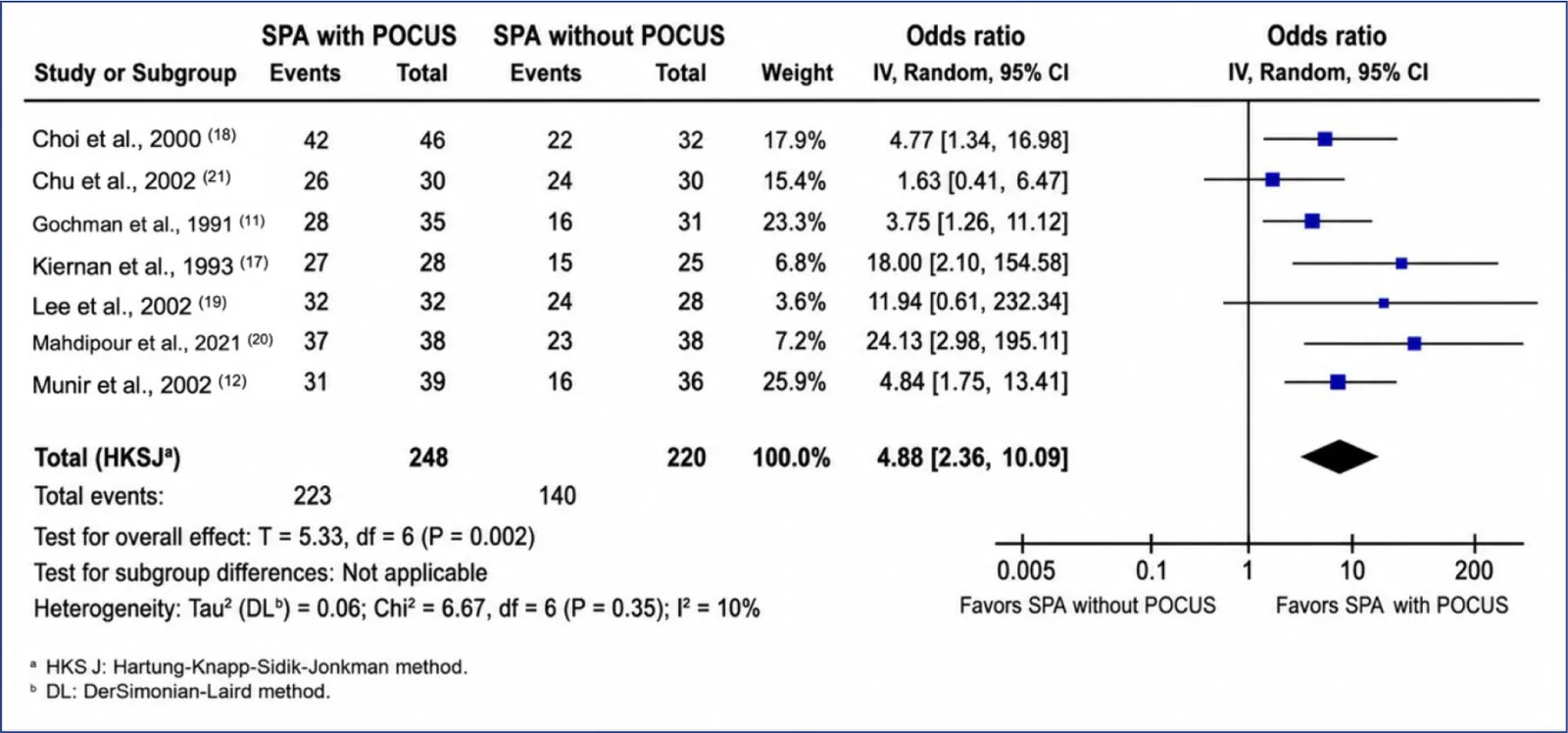
Forest plot showing the overall success rate of SPA, comparing the POCUS group with the control group. HKSJ, Hartung-Knapp-Sidik-Jonkman; REML, restricted maximum likelihood; df, degrees of freedom.

The pooled analysis demonstrated significantly higher odds of success for POCUS-guided SPA than for tradi-tional SPA (OR = 4.88; 95% CI: 2.36-10.09; *p* < 0.0001; I^^2^^ = 10%). A forest plot (Figure 2) shows that most studies favored the POCUS approach, with the odds ratio being highest for the study conducted by Mahdipour et al.^**(^21^)**^: 24.13; 95% CI: 2.98, 196.11. The overall success rate for POCUS-guided SPA was 89.9%, compared with 63.6% for the control (traditional SPA), representing a relative difference of approximately 41%.

**Figure 3:**
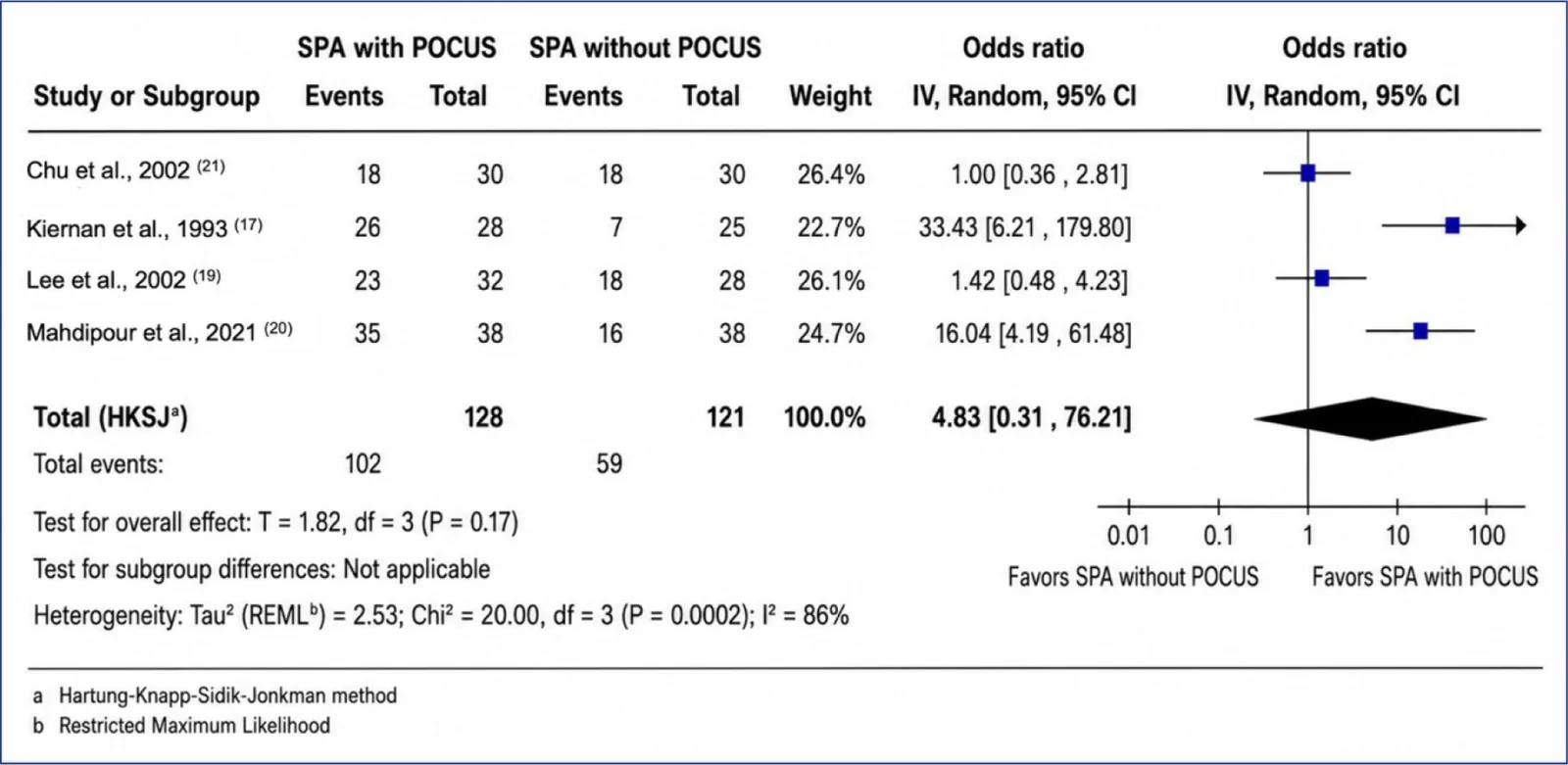
Forest plot of SPA success at first attempt, comparing the POCUS group with the control group.

HKSJ, Hartung-Knapp-Sidik-Jonkman; REML, restricted maximum likelihood; df, degrees of freedom.

**Figure 4:**
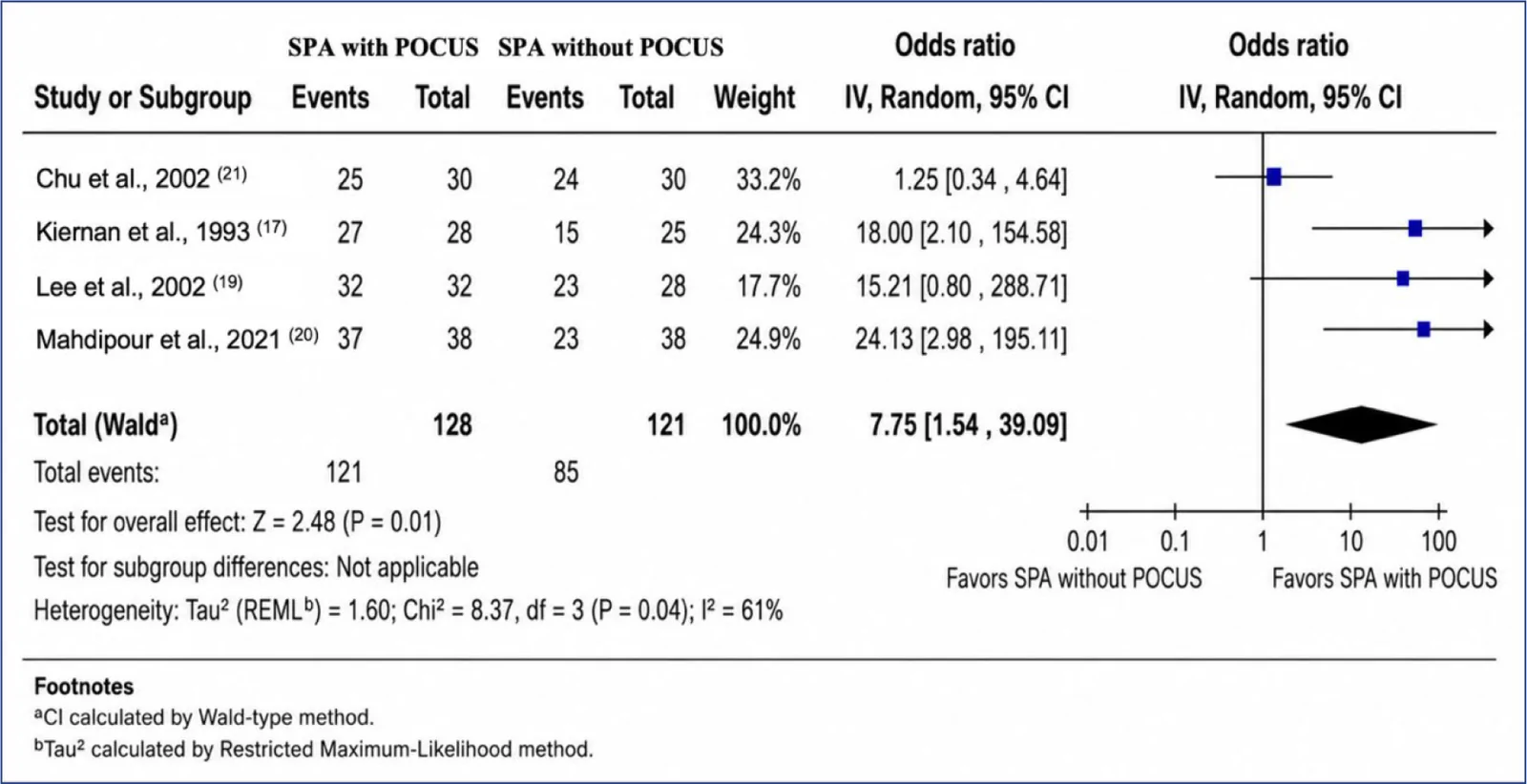
Forest plot of SPA success up to two attempts, comparing the POCUS group with the control group. REML, restricted maximum likelihood; df, degrees of freedom.

A secondary analysis revealed that POCUS guidance does not have a significant impact on first-attempt odds of success (OR = 4.83; 95% CI: 0.31-76.21; *p* = 0.17; I^^2^^ = 86%). That analysis also showed that there was high heterogeneity among the studies (I^^2^^ = 86%), suggesting substantial variation in the results (Figure 3), as further discussed below. There was a statistically significant dif-ference in the odds of success within up to two attempts (OR = 7.75; 95% CI: 1.54-39.09; *p* = 0.01; I^^2^^ = 61%), further supporting the superiority of POCUS in this context (Figure 4). Leave-one-out sensitivity analysis was not performed due to the low I^^2^^ indicated.

**Figure 5:**
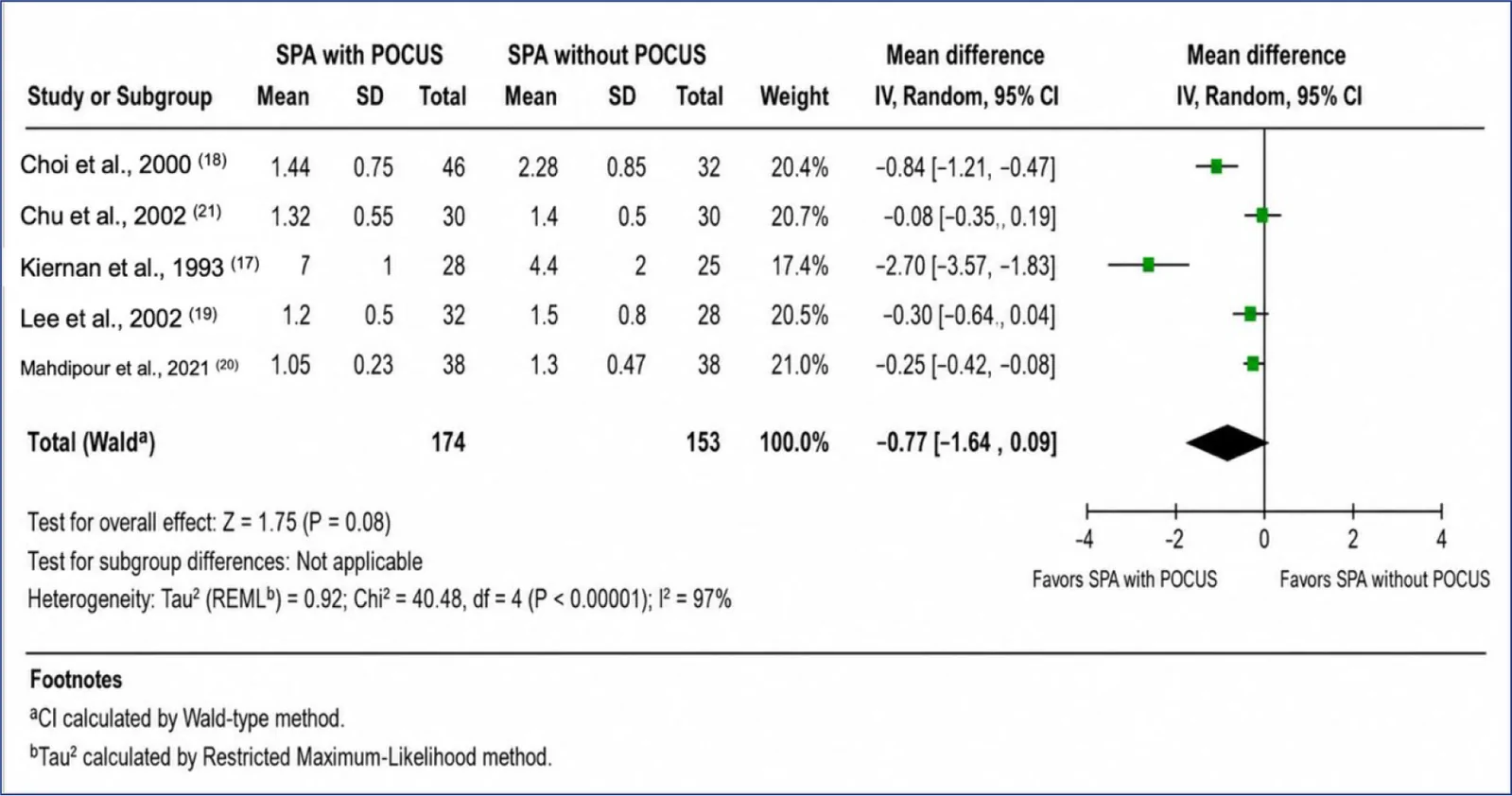
Forest plot of mean number of attempts to collect urine comparing the POCUS group with the control group. REML, restricted maximum likelihood; df, degrees of freedom.

### Mean number of attempts

POCUS guidance did not have a significant impact on reducing the number of attempts, with a mean difference (MD) of -0.77 (95% CI: -1.64 to 0.09; *p* = 0.08; I^^2^^ = 97%). When the study conducted by Kiernan et al.^**(^18^)**^ was excluded, the leave-one-out sensitivity analysis showed a significant benefit of POCUS guidance (MD = -0.35; 95% CI: -2.02 to 0.09; I^^2^^ = 73%).

### Sensitivity analysis

**Figure 6:**
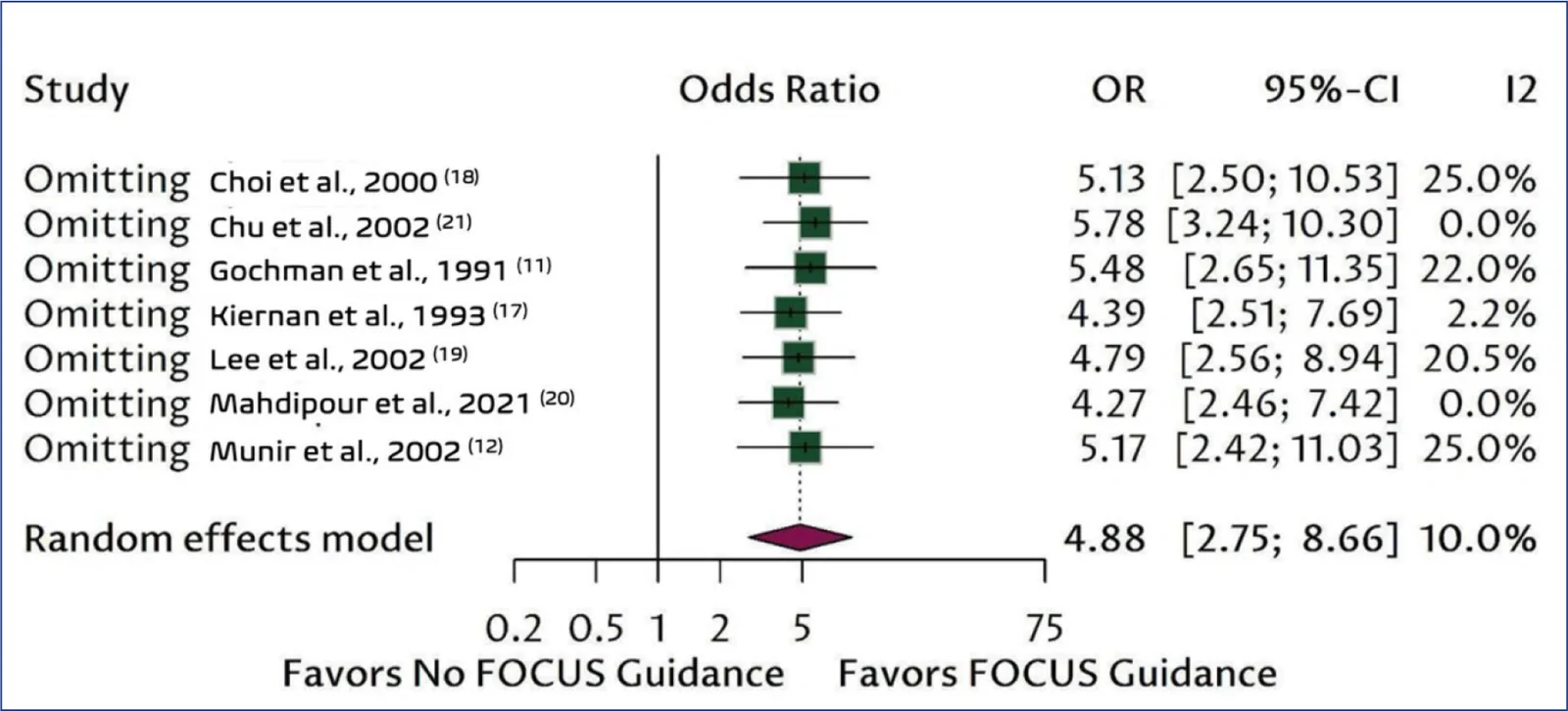
Sensitivity analysis of the overall success rate, examining the stability of the pooled overall success rate for suprapubic bladder aspiration. By sequentially omitting one study at a time, the analysis evaluated the impact of each individual study on the combined estimate, aiming to detect potential outliers or influential studies.

**Figure 7:**
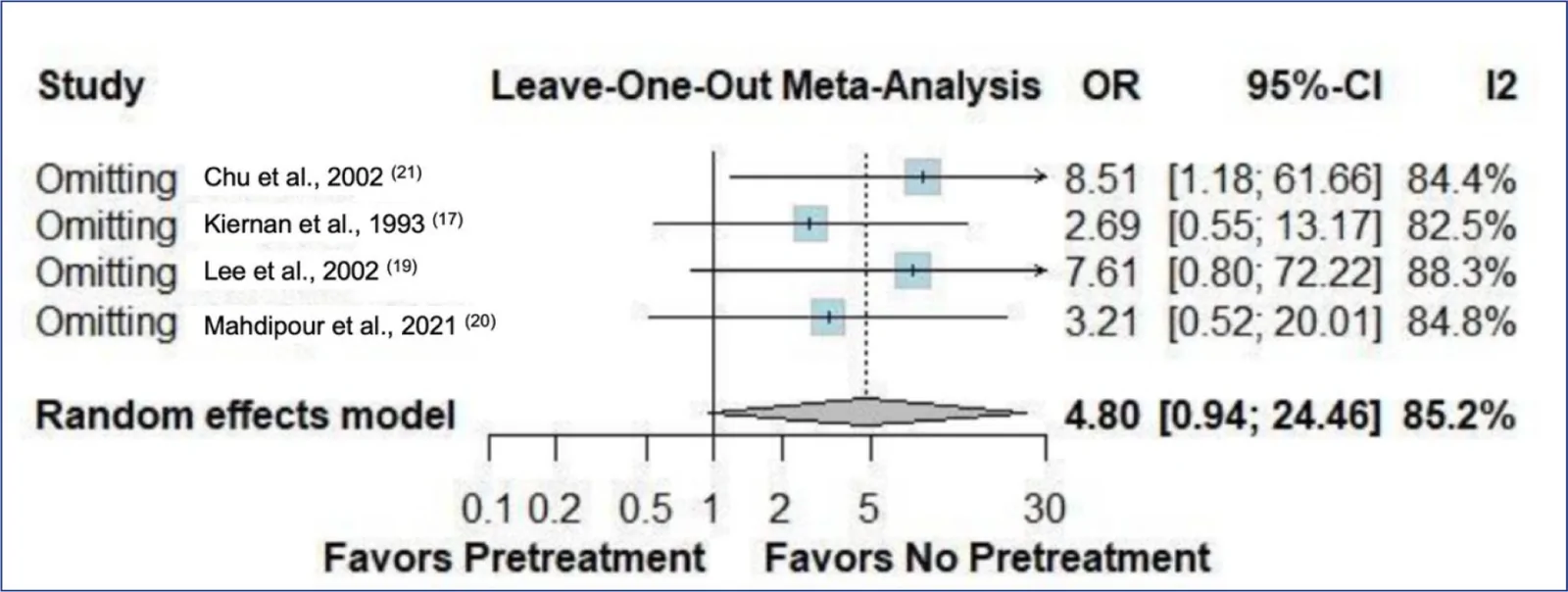
Sensitivity analysis of success at first attempt, evaluating the robustness of the pooled estimate for first-attempt success in suprapubic bladder aspiration. Each iteration involves the exclusion of one study at a time to examine its influence on the overall effect size. The analysis helps identify whether any single study disproportionately affects the pooled outcome.

**Figure 8:**
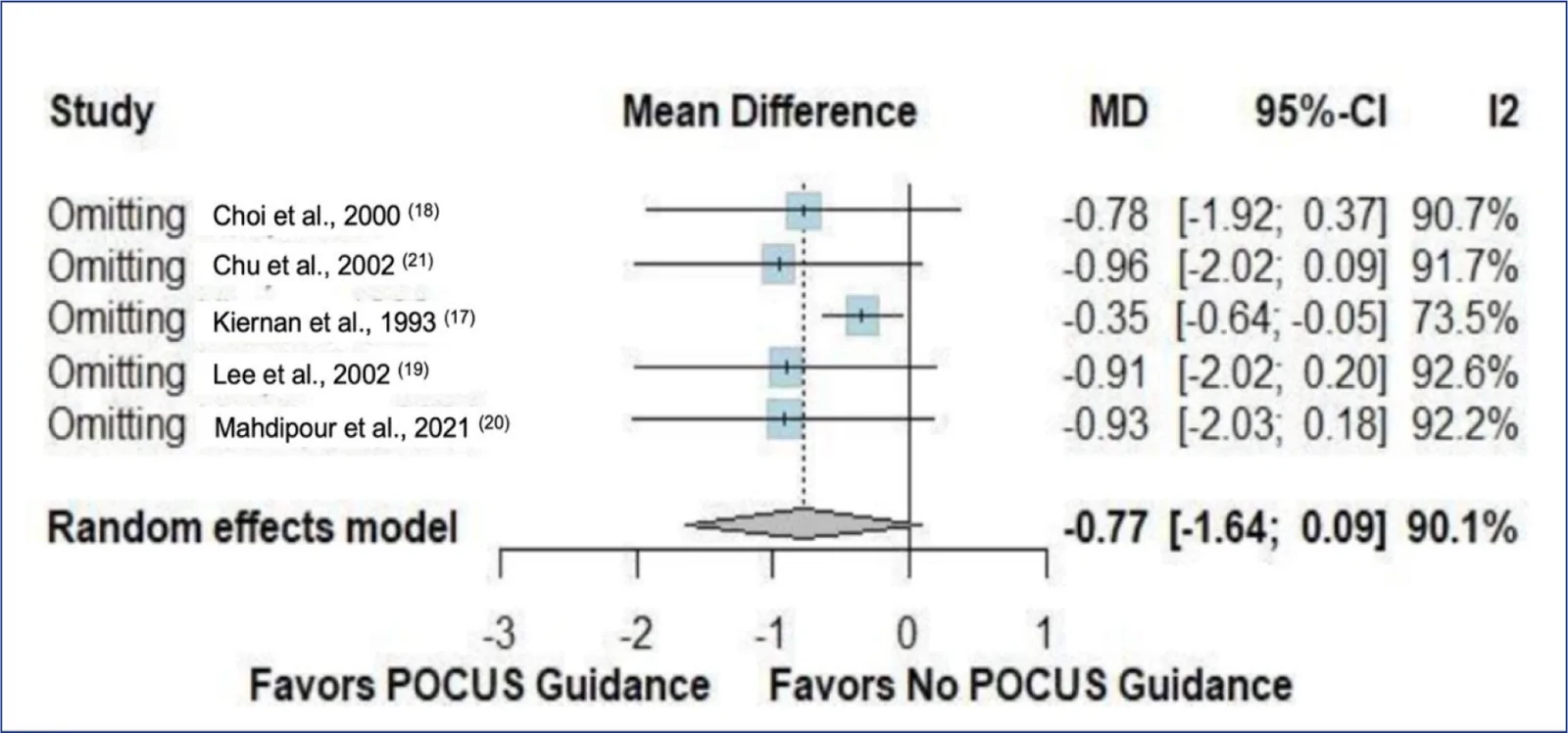
Sensitivity analysis of the mean number of attempts, assessing the robustness of the pooled mean number of attempts across the studies included. Variations in effect estimates were explored by sequentially excluding individual studies.

A sensitivity analysis performed for the overall suc-cess rate showed that omitting the studies conducted by Chu et al.^**(^22^)**^ and Mahdipour et al.^(^21^)^ had a significant influence on heterogeneity, with ORs of 5.78 ( 95% CI: 3.24-10.30; I^^2^^ = 0.0%) and 4.27 (95% CI: 2.46-7.42; I^^2^^ = 0.0%), respectively (Figure 6). A sensitivity analysis for first-attempt success did not find a specific study respon-sible for the heterogeneity (Figure 7). A sensitivity analysis for mean number of attempts identified the Kiernan et al. study^**(^18^)**^ as the primary source of heterogeneity, and exclusion of that study resulted in the achievement of statistical significance (MD = -0.35; 95% CI: -2.02 to 0.09; I^^2^^ = 73%). Nonetheless, high heterogeneity persisted across studies (Figure 8).

### Risk of bias assessment

**Figure 9:**
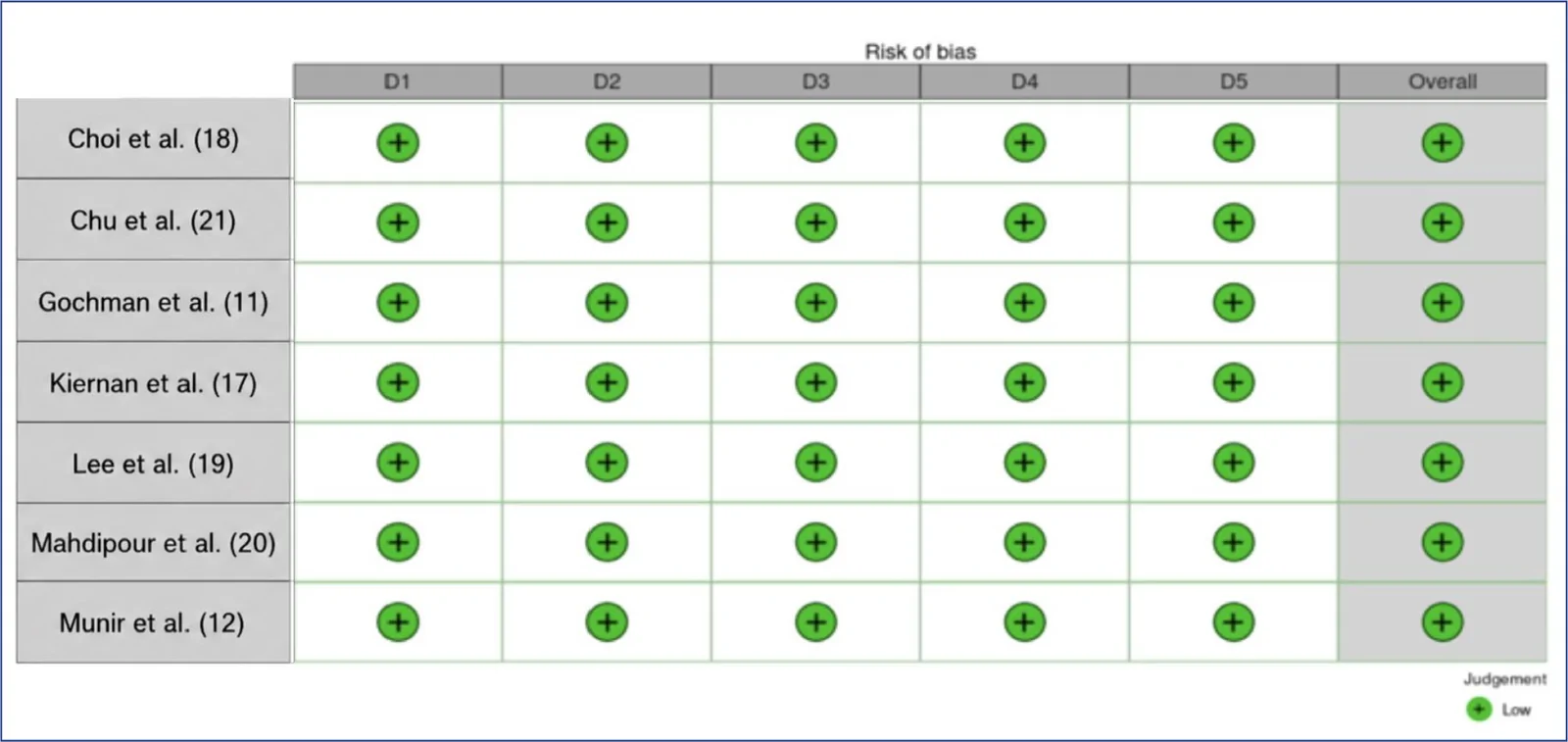
Risk of bias summary, providing a visual overview of the risk of bias across the included studies, evaluating domains such as randomization process, deviations from intended interventions, missing outcome data, measurement of the outcome, and selection of the result reported.

The risk of bias across the five domains assessed for RCTs (selection, performance, detection, attrition, and reporting biases), evaluated by using the RoB 2 tool, was low and showed concordance between the two evaluators (E.F.M and L.B.S.C.), as illustrated in Figure 9.

## DISCUSSION

In this systematic review and meta-analysis, the pooled results of 468 patients demonstrated a significant improvement in the success rate of SPA due to POCUS guidance in pediatric patients. Overall, considering sum-marized raw data across studies, the success rate was 89.9% with POCUS and 63.6% without, representing a relative difference of approximately 41%. Success at first attempt occurred in 102 (79.7%) of the 128 cases in which POCUS was employed, compared with 59 (48.8%) of the 121 cases in which it was not. These findings indicate that using POCUS may improve the efficiency of SPA and reduce patient distress associated with the procedure by reducing the number of attempts needed. In addition to these benefits, POCUS offers practical advantages such as estimating bladder dimensions, confirming adequate bladder filling, and enabling real time visualization of the needle during the procedure, all of which contribute to a higher rate of procedural success.

Despite the benefits of POCUS in the overall suc-cess rate of SPA, some important considerations must be addressed. The first is the variability in operator experience, which may influence success rates and adoption of the method. Studies conducted in Brazil and Saudi Arabia, highlight a significant gap in medi-cal education regarding POCUS training. A study conducted in Saudi Arabia showed that only 21.4% of final-year medical students had received formal POCUS training, revealing a deficiency in ultrasound proficiency among future physicians^**(^23^)**^. Similarly, research involving members of the Brazilian Society of Nephrology revealed that the majority (64%) of participants did not have the opportunity to develop ultrasound examination skills during their medical training^(^24^)^. Despite the well-documented benefits of POCUS, few medical programs offer structured training in this skill, emphasizing the urgent need to integrate ultrasound education into medical cur-ricula to enhance the diagnostic and procedural capabilities of physicians^(^25^)^. In addition, there were notable methodological discrepancies among the trials included. The fact that only one study-that conducted by Mahdipour et al.^**(^21^)**^-explicitly re-ported which health care professional performed each procedure limited our ability to assess the influence of operator expertise.

Availability is another important factor that may limit the widespread implementation of POCUS in SPA. While ultrasound devices are becoming more ac-cessible, their availability remains inconsistent across different health care settings, particularly in low-resource settings^**(^13^)**^. Institutions with limited funding may lack portable ultrasound machines or may not have enough trained personnel to ensure real-time imaging during SPA procedures. In contrast, traditional (blind) SPA, although less precise, does not require additional equipment. Therefore, cost-effectiveness analyses comparing POCUS-guided SPA with alternative urine collection methods are warranted in order to guide policy decisions and optimize resource allocation.

This study has some limitations that should be considered. The majority of the studies included were published between 1991 and 2002, with the notable exception of that conducted by Mahdipour et al.^**(^21^)**^, which was published in 2021. Given the advances in technology and widespread use of POCUS, including integration of artificial intelligence, generalizability and contemporary applicability may be limited^**(^26^)**^. However, these technological innovations may have further im-proved SPA success rates, resulting in an underestimation of the impact of POCUS in our study. Bladder volume is also an important source of variability across studies, given that it represents an essential determinant of proce-dural success. Munir et al.^**(^12^)**^ demonstrated that the SPA success rate reaches nearly 90% when bladder volume exceeds 10 mL on ultrasound, highlighting the need for adequate bladder filling before the procedure. However, the remaining studies included in this meta-analysis did not apply a standardized bladder volume threshold, which may have contributed to the heterogeneity observed in diagnostic accuracy. Future methodologically rigorous RCTs should adopt consistent definitions and measure-ment protocols for bladder volume to more accurately evaluate its influence on the performance and success of ultrasound-guided SPA.

The accuracy of POCUS for SPA in infants depends on the technical capabilities of the ultrasound system. Older-generation machines with low resolution, analog interfaces, and minimal pediatric optimization likely increase reliance on operator skill and contribute to increasing the number of failed or uncertain punctures. In contrast, newer digital systems, introduced in the early 2000s, offer better image quality and usability, improving the effectiveness and ease of the procedure. In comparison, the device used by Gochman et al.^**(^11^)**^ in their study published in 1991 - the ScanMate II ultrasound system - was limited to a 60° scan angle, and a 10-cm depth range. In contrast, Mahdipour et al.^**(^21^)**^, in their 2021 study, used the Ultrasonix plat-form, which offered significantly higher resolution and advanced technologies like SonixDAQ, enabling over 10,000 frames per second and photoacoustic imaging. These technological developments appear to improve image clarity and facilitate real-time targeting, as well as potentially reducing operator dependency during pro-cedures such as SPA. Enhanced bladder visualization, including in small infants, could potentially increase procedural accuracy. Such evolving capabilities may, in part, contribute to the heterogeneity observed across studies as ultrasound technology has advanced over time.

Heterogeneity was observed, particularly in first-attempt success rates (I^^2^^ = 86%; *p* > 0.001), potentially due to variations in operator expertise, ultrasound equip-ment, and reason for aspiration. For example, Gochman et al.^**(^11^)**^ and Kiernan et al.^**(^18^)**^ used the judgement of the medical team to determine the reason for aspiration, whereas Chu et al.^**(^22^)**^ used objective criteria: a urine sample testing positive for leukocyte esterase or nitrites; or the presence of leukocytes (> 5 mm^3^) on microscopic examination. Although the mean number of attempts needed to achieve a successful SPA was lower among the POCUS-guided procedures than among the traditional procedures, statistical significance was not reached. This outcome appears to be influenced by substantial hetero-geneity, primarily driven by a single outlier study - that conducted by Kiernan et al.^(^18^)^ - the results of which were markedly divergent from those of the other stud-ies included in this review. Sensitivity analysis with the leave-one-out method suggests that exclusion of this study would have resulted in a statistically significant difference favoring POCUS, underscoring the considerable impact of the outlier on the overall findings.

Although the present meta-analysis provides valuable insights, it is important to acknowledge that most of the studies included are relatively dated and exhibit considerable methodological heterogeneity, which may limit the generalizability and current applicability of the results. Future research should prioritize the development of well-designed, contemporary RCTs that address these limitations. In particular, key methodological aspects such as operator expertise, ultrasound technology, and bladder volume at the time of assessment should be standardized or systematically evaluated to ensure that the findings are more reliable and reproducible across studies.

## 5. CONCLUSION

This meta-analysis demonstrates the superiority of POCUS-guided SPA over traditional blind SPA in terms of the overall success rates of the procedure in pediatric patients. Compared with blind SPA, POCUS-guided SPA remains a more successful approach for obtaining a sterile urine sample in children. Despite the heterogeneity observed among the studies included in this review, the overall evidence supports the in-tegration of POCUS into routine pediatric practice.

## Data Availability

Not applicable

## References

[1] Mattoo TK, Shaikh N, Nelson CP. (2021). Contemporary Manage-ment of Urinary Tract Infection in
Children. Pediatrics.

[2] Greenhow TL, Hung YY, Herz AM (2014). The changing epidemiology of serious bacterial infections in
young infants. Pediatr Infect Dis J.

[3] Kuppermann N, Dayan PS, Levine DA (2019). A Clinical Predic-tion Rule to Identify Febrile Infants 60 Days
and Younger at Low Risk for Serious Bacterial Infections. JAMA Pediatr.

[4] Shaikh N, Morone NE, Bost JE (2008). Prevalence of urinary tract infection in childhood: a
meta-analysis. Pediatr Infect Dis J.

[5] Ladomenou F, Bitsori M, Galanakis E. (2015). Incidence and morbidity of urinary tract infection in a
prospective cohort of children. Acta Paediatr.

[6] Diviney J, Jaswon MS. (2021). Urine collection methods and dipstick testing in
non-toilet-trained children. Pediatr Nephrol.

[7] ’t Hoen LA, Bogaert G, Radmayr C (2021). Update of the EAU/ ESPU guidelines on urinary tract infections in
children. J Pediatr Urol.

[8] SUBCOMMITTEE ON URINARY TRACT INFECTION (2016). Reaffirmation of AAP Clinical Practice Guideline: The Diagnosis
and Management of the Initial Urinary Tract Infection in Febrile Infants and
Young Children 2-24 Months of Age. Pediatrics.

[9] Baumann BM, McCans K, Stahmer SA (2007). Caregiver and health care provider satisfaction with volumetric
bladder ultrasound. Acad Emerg Med.

[10] Díaz-Gómez JL, Mayo PH, Koenig SJ. (2021). Point-of-Care Ultrasonography. N Engl J Med.

[11] Gochman RF, Karasic RB, Heller MB. (1991). Use of portable ultrasound to assist urine collection by
suprapubic aspiration. Ann Emerg Med.

[12] Munir V, Barnett P, South M. (2002). Does the use of volumetric bladder ultrasound improve the success
rate of suprapubic aspiration of urine?. Pediatr Emerg Care.

[13] Hassanzadeh Rad A, Badeli H. (2017). Point-of-Care Ultrasonography: Is It Time Nephrologists Were
Equipped With the 21th Century’s Stethoscope?. Iran J Kidney Dis.

[14] Sterne JAC, Savovic´ J, Page MJ (2019). RoB 2: a revised tool for assessing risk of bias in randomised
trials. BMJ.

[15] Review Manager (RevMan) [Computer program] (2020). https://train-ing.cochrane.org.

[16] RStudio: Integrated Development Environment for R [Posit
Software] (2024). http://www.posit.co/.

[17] R Core Team (2024). Vienna.

[18] Kiernan SC, Pinckert TL, Keszler M. (1993). Ultrasound guidance of suprapubic bladder aspiration in
neonates. J Pediatr.

[19] Choi BM, Kim KB, Lee HH (2001). The Use of Ultrasonography in the Suprapubic Bladder Aspiration
in Neonates. J Korean Soc Neonatol.

[20] Lee JW, Park S, Cho SJ (2002). Suprapubic Bladder Aspiration Assisted by
Ultrasound. J Korean Soc Pediatr Nephrol.

[21] Mahdipour S, Saadat SNS, Badeli H (2021). Strengthening the success rate of suprapubic aspiration in
infants by integrating point-of-care ultrasonography guidance: A
parallel-randomized clinical trial. PloS One.

[22] Chu RWP, Wong YC, Luk SH (2002). Comparing suprapubic urine aspiration under real-time ultrasound
guidance with conventional blind aspiration. Acta Paediatr.

[23] Nassar SM, Almubrik SA, Alyahya L (2024). Perception, Knowl-edge, Indications, and Future Prospects of
Point-of-Care Ultra-sound Among Medical Students in Saudi
Arabia. Cureus.

[24] Bastos MG, Vieira AL, Nascimento MM do (2020). Point-of-care ultrasonography in nephrology: a cross-sectional
national survey among Brazilian nephrologists. Braz J Nephrol J Bras Nefrol.

[25] Gold D, Levine M, Hsu D (2021). Design of a point-of-care ultra-sound curriculum for pediatric
emergency medicine fellows: A Delphi study. AEM Educ Train.

[26] Venkatayogi N, Gupta M, Gupta A (2023). From Seeing to Know-ing with Artificial Intelligence: A Scoping
Review of Point-of-Care Ultrasound in Low-Resource Settings. Appl Sci.

